# Binding Success and Failure: Evidence for the Spontaneous Integration of Perceptual Features and Object Evaluations

**DOI:** 10.3389/fpsyg.2012.00581

**Published:** 2012-12-27

**Authors:** Bernhard Hommel, André W. Keizer

**Affiliations:** ^1^Cognitive Psychology Unit, Leiden Institute for Brain and Cognition, Leiden UniversityLeiden, Netherlands; ^2^Department of Psychology, Cognitive Neuroscience Group, University of AmsterdamAmsterdam, Netherlands

**Keywords:** feature binding, affective binding, object file, event integration, emotion

## Abstract

Humans represent perceptual events in a distributed, feature-specific fashion, which calls for some sort of feature integration. It has been suggested that processing an event leads to the creation of a temporary binding of the corresponding feature codes – an object file. Here we show that object files do not only comprise of perceptual feature codes but also include codes that reflect evaluations of the perceptual event.

## Introduction

Humans represent the events they perceive in a distributed fashion, which calls for some sort of feature integration. Kahneman et al. (1992) have argued that people bind the cognitive codes of event features into temporary object files. They demonstrated that participants respond particularly fast and accurately to repeated stimuli if these also appear in the same location, suggesting that the first encounter led to the binding of shape and location codes. Moreover, repeating one or more features of a stimulus but alternating others impairs performance (Hommel, 1998), suggesting that feature-repetition leads to the automatic retrieval of the just-created binding, which interferes with processing the present feature combination if it differs from the previous one (Hommel, 2004). Indeed, repeating one of two features of a visual stimulus reactivates the cortical area coding for the non-repeated feature (Keizer et al., 2008). Research on object files has mainly focused on the binding of perceptual feature codes. However, feature codes are no copies of external events but brain responses to those events, which raises the question whether other, non-perceptual responses become part of an object file as well. Here we investigated whether object files also contain information about people’s evaluative responses to a stimulus.

**Figure 1:**
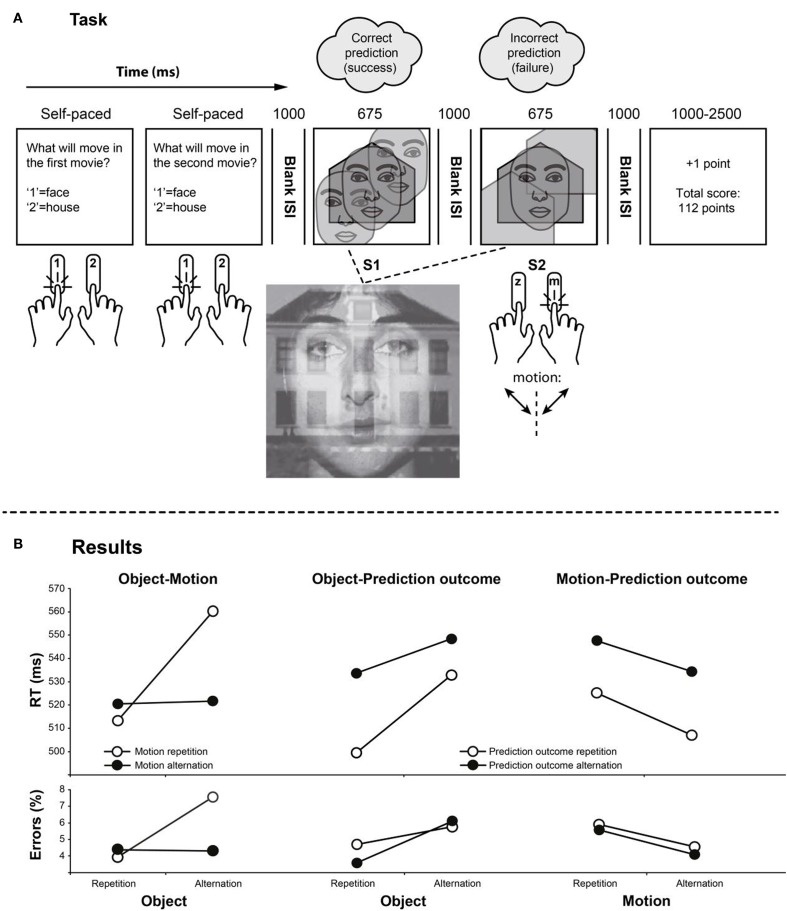
**(A)** Sequence of events, from left to right. **(B)** Reaction times (RTs) and error percentages as a function of object repetition/alternation and motion repetition/alternation (leftmost panels), object repetition/alternation and prediction-outcome repetition/alternation (middle panels), and motion repetition/alternation and prediction-outcome repetition/alternation (rightmost panels).

As in Keizer et al. (2008), we presented participants with pairs of stimuli in each trial, a prime (S1) followed by a probe (S2; see Figure 1A). Both stimuli consisted of blends of a face and a house, and either the face or the house moved diagonally up and down. Participants did not respond to S1 but categorized the moving object’s motion direction (top-left/bottom-right or top-right/bottom-left). This task allowed for the orthogonal repetition and alternation of the moving object and the direction in which it moved. The integration of moving object and motion upon processing of S1 was expected to yield an interaction of the two repetition effects, with a pattern that indicates worse performance if one of the two features repeats while the other alternates (Keizer et al., 2008).

Evaluative responses to the two stimuli were induced by making participants believe that the sequence of the objects that moved followed a consistent pattern across trials and having them predict whether a face or house would move on S1 and on S2. Encountering a stimulus that meets the prediction was considered to evoke a positive evaluation (success), while stimuli not meeting the prediction were thought to evoke a negative evaluation (failure). The question was whether the effect of repeating the type of evaluative response (in trials where the two predictions happened to be both correct or both incorrect) vs. alternating the evaluative response (in trials where just one prediction happened to be correct) would interact with the effect of repeating vs. alternating the type of object being moved and/or the type of motion. If so, this would suggest that codes related to visual event features are integrated with codes representing event-related success and failure.

## Materials and Methods

Twenty-four students participated for course credit or pay. They judged the motion direction shown in S2 by pressing a left vs. right key of a computer keyboard. After predictions were made, a trial would start with a 1000-ms blank interval. Then S1 appeared for 675 ms, followed by a 1000-ms blank interval. Thereafter, S2 appeared for 675 ms, followed by another 1000-ms blank interval.

As in Keizer et al. (2008), visual stimuli were composed by superimposing luminance-matched grayscale front-view photographs of male and female faces and of houses. The house-face combinations for the 240 trials were constructed by randomly drawing from eight possible houses and faces, except that all four combinations of repeating vs. alternating the moving object (face or house) were equally likely. The face image and the house images for a given trial were randomly selected from the set of eight face images and eight house images, and they were always the same for S1 and S2.

Before the stimuli were presented, participants predicted whether a face or a house would move on S1 and S2 by pressing the “1” or “2” key on a computer keyboard (counterbalanced), respectively. They received points for each correct prediction (i.e., 0–2 per trial) and the number of earned points, together with a running total, was presented at the end of each trial. The three highest-scoring participants received an extra of 5€ after the experiment was completed.

## Results

The RTs and error rates for the response to S2 were analyzed. Trials in which RTs deviated more than 2 SDs from the mean were excluded. The remaining data were aggregated according to whether the moving object was the same in S1 and S2 (object repetition) or different (object alternation), the direction of the movement was the same for S1 and S2 (movement repetition) or different (movement alternation), and whether the outcomes of the two predictions in each trial were the same (two times success or two times failure; a prediction outcome repetition) or different (one failure and one success; a prediction outcome alternation). RTs and error rates from the resulting eight design cells were entered into ANOVAs with three corresponding factors:repetition (vs. alternation) of moving object, motion, and prediction outcome (see Figure 1B; Table 1).

**Table 1:** **Means of mean reaction times and SD for responses (RT; in ms) and percentages of errors (PE) for responses to stimulus 2, as a function of the repetition vs. alternation of motion direction, moving object, and reward**.

Motion	Repeated				Alternated			
Moving object	Repeated		Alternated		Repeated		Alternated	
Prediction outcome	RT (SD)	PE (SD)	RT (SD)	PE (SD)	RT (SD)	PE (SD)	RT (SD)	PE (SD)
Repeated	497 (53)	5 (6)	553 (70)	7 (6)	502 (56)	5 (7)	513 (60)	5 (6)
Alternated	529 (65)	3 (4)	567 (56)	8 (6)	539 (62)	4 (6)	530 (61)	4 (5)

In RTs, responses were faster upon the repetition of moving object, *F*(23,1) = 53.2, MSE = 523.9, *p* < 0.001, and prediction outcome, *F*(23,1) = 66.2, MSE = 448.0, *p* < 0.001, and slower if the type of motion was repeated, *F*(23,1) = 12.8, MSE = 928.5, *p* < 0.005. More importantly for our purposes, interactions were obtained for moving object and motion, *F*(1,23) = 106.4, MSE = 236.2, *p* < 0.001, and moving object and prediction outcome, *F*(1,23) = 7.0, MSE = 599.1, *p* < 0.05; separate analyses confirmed that the latter did not depend on whether the outcomes were positive or negative.

Error rates followed the same pattern: performance was more accurate if the moving object repeated, *F*(1,23) = 10.4, MSE = 14.6, *p* < 0.005, an effect that interacted with the repetition of motion, *F*(1,23) = 7.5, MSE = 22.1, *p* < 0.05. The only exception was the interaction of moving object and prediction outcome, which showed the opposite pattern of the RTs – a more pronounced object repetition-alternation effect with prediction alternation. However, the corresponding interaction was far from significance, *F*(1,23) = 1.35, MSE = 24.15.6, *p* > 0.25.

## Discussion

We were able to replicate the well-known observation of worse performance if a visual feature is repeated while another alternates (Hommel, 1998), suggesting that participants spontaneously integrated the codes of these features – the type of moving object and motion direction in our case. In addition to the more interesting interactions, we also obtained main effects of all three experimental factors. In the cases of object repetition and prediction outcome repetition, the underlying pattern is rather straightforward: alternations of features can be suspected to create neural conflict between the present and the previous feature values (Kühn et al., 2011), which slows down object identification. In the case of motion, however, alternations produced faster, rather than slower responses. Even though the interaction with object repetition (see Figure 1B) makes the interpretation difficult, we speculate that the exposure to a repeated motion pattern over 675 ms might have resulted in motion adaptation (Ölveczky et al., 2007), which impaired the processing of objects moving into the same direction. This need not have prevented the standard feature-repetition benefit but it might have overshadowed this effect in the data.

More importantly, however, our findings demonstrate that participants coded their successes and failures in predicting the motion direction of the two visual stimuli, and integrated these codes with codes representing the moving object. Interestingly, this integration seemed to be selective for the object feature that the prediction was referring to, while there was no evidence that motion direction interacted with prediction outcomes. This might suggest that object files do not simply lump together all information that relates to a given object but, rather, consist of a complex, multi-level representational structure (Hommel, 2004). However, it is also possible that all available information was actually integrated but only partially retrieved while processing S2. Hence, it might be that control processes modulate stimulus-driven retrieval of information in such a way that only relevant feature codes are retrieved to a degree that allows affecting behavior (e.g., Keizer et al., 2010).

Also of importance for the purpose of the present study, the interaction between the repetition of object features and the repetition of the outcomes of object-related judgments suggests that codes referring to the physical features of object are integrated with codes referring to the evaluation of objects or object-related aspects. In other words, object files seem to allow for the evaluative “tagging” of visual feature codes. It is interesting to consider how “affective” or “emotional” these evaluative codes actually are. On the one hand, one might consider them some kind of “somatic markers” (Damasio, 1994) that relate to and represent the emotional experience one had when creating them. If so, reactivating an evaluative code in the process of a stimulus-induced retrieval of object information might lead to the recall or simulation of the emotional state one was in when having experienced success or failure with regard to this particular object. In this case, evaluative codes may actually be considered “affective markers.” On the other hand, however, it is also possible that evaluative codes only indicate successes and failures without necessarily revoking any related emotional state. In a recent study, Eder et al. (submitted) provided evidence that actions are not only integrated with representations of their affective consequences (Eder and Hommel, in press) but that these representations have two different kinds of effect on action control: a directive function when selecting responses in a stimulus-driven forced-choice task and (in addition) an incentive function when selecting freely chosen actions. It is possible that the former is based on the mere information whether a particular response will or will not produce positive outcomes while the latter relies on a simulation of the expected affective state in the sense of Damasio (1994). If so, the present study might be taken to speak more to the representations underlying the directive function of evaluation-related outcome representations.

## Conflict of Interest Statement

The authors declare that the research was conducted in the absence of any commercial or financial relationships that could be construed as a potential conflict of interest.

## References

[B1] Damasio A. R. (1994). Descartes’ Error: Emotion, Reason, and the Human Brain. New York: Grosset/Putnam

[B2] Eder A. B. Hommel B. (in press). Anticipatory control of approach and avoidance: an ideomotor approach. Emotion Review.

[B3] Hommel B. (1998). Event files: evidence for automatic integration of stimulus-response episodes. Vis. cogn. 5, 183–216 10.1080/713756773

[B4] Hommel B. (2004). Event files: feature binding in and across perception and action. Trends Cogn. Sci. (Regul. Ed.) 8, 494–500 10.1016/j.tics.2004.08.007 15491903

[B5] Kahneman D. Treisman A. Gibbs B. J. (1992). The reviewing of object files: object-specific integration of information. Cogn. Psychol. 24, 175–219 10.1016/0010-0285(92)90007-O 1582172

[B6] Keizer A. W. Nieuwenhuis S. Colzato L. S. Theeuwisse W. Rombouts S. A. R. B. Hommel B. (2008). When moving faces activate the house area: an fMRI study of object file retrieval. Behav. Brain Funct. 4, 50 10.1186/1744-9081-4-50 18945346 PMC2577093

[B7] Keizer A. W. Verment R. Hommel B. (2010). Enhancing cognitive control through neurofeedback: a role of gamma-band activity in managing episodic retrieval. Neuroimage 49, 3404–3413 10.1016/j.neuroimage.2009.11.023 19925870

[B8] Kühn S. Keizer A. Colzato L. S. Rombouts S. A. R. B. Hommel B. (2011). The neural underpinnings of event-file management: evidence for stimulus-induced activation of, and competition among stimulus-response bindings. J. Cogn. Neurosci. 23, 896–904 10.1162/jocn.2010.21485 20377359

[B9] Ölveczky B. P. Baccus S. A. Meister M. (2007). Retinal adaptation to object motion. Neuron 56, 689–700 10.1016/j.neuron.2007.09.030 18031685 PMC2117331

